# Epigenetic inactivation of the extracellular matrix metallopeptidase *ADAMTS19* gene and the metastatic spread in colorectal cancer

**DOI:** 10.1186/s13148-015-0158-1

**Published:** 2015-12-02

**Authors:** Sergio Alonso, Beatriz González, Tatiana Ruiz-Larroya, Mercedes Durán Domínguez, Takaharu Kato, Akihiro Matsunaga, Koichi Suzuki, Alex Y. Strongin, Pepita Gimènez-Bonafé, Manuel Perucho

**Affiliations:** Institute of Predictive and Personalized Medicine of Cancer (IMPPC), Institut d’investigació en ciéncies de la salut Germans Trias I Pujol, (IGTP), Campus Can Ruti, 08916 Badalona, Barcelona Spain; Sanford Burnham Prebys Medical Dicovery Institute, 10901 N. Torrey Pines Rd. La Jolla, San Diego, CA 92037 USA; Cancer Genetics Laboratory, IBGM-CSIC, University of Valladolid, Valladolid, 47005 Spain; Department of Surgery, Saitama Medical Center, Jichi Medical University, 1-847, Amanuma-cho, Omiya-ku, Saitama, 330-8503 Japan; Departament de Ciències Fisiològiques II, Campus Ciènces de Salut de Bellvitge, IDIBELL, University of Barcelona, Barcelona, 08907 Spain; Institució Catalana de Recerca i Estudis Avançats (ICREA), Catalan Institution for Research and Advanced Studies. Pg. Lluís Companys 23, 08010 Barcelona, Spain; Institute of Predictive and Personalized Medicine of Cancer (IMPPC), Carretera de Can Ruti, Cami de les Escoles S/n, 08916 Badalona, Barcelona Spain

**Keywords:** ADAMTS, Methylation, Matrix metallopeptidases, Gastrointestinal cancer, Ovarian cancer, MS-AFLP

## Abstract

**Background:**

*ADAMTS19* encodes a member of the ADAMTS (a disintegrin and metalloproteinase domain with thrombospondin motifs) protein family with emerging roles in carcinogenesis and metastasis. ADAMTS shares several distinct protein modules including a propeptide region, a metalloproteinase domain, a disintegrin-like domain, and a thrombospondin type 1 (TS) motif. In a previous work, we found *ADAMTS19* frequently hypermethylated in colorectal cancer (CRC). We explored the association of methylation with tumor genotype and phenotype.

**Results:**

The methylation status of the CpG island in the promoter of *ADAMTS19* was determined in 252 colorectal, 65 pancreatic, 33 breast and 169 ovarian primary tumors, 70 CRC metastases, and 10 CRC cell lines. Tumor-specific methylation of *ADAMTS19* was significantly more frequent in gastrointestinal than in gynecological cancers (odds ratio (OR) = 2.9, confidence interval (CI) = (1.9–4.7), *p =* 5.2 × 10^−7^) and was independent of the methylation of adjacent *loci* in CRC. Hypermethylation associated with CRC with mutated *BRAF* oncogene (OR = 10.1, CI = (3.1–42.9), *p =* 6.3 × 10^−6^) and with the mucinous phenotype in CRC (OR = 2.1, CI = (1.1–4.1), *p =* 0.023) and ovarian cancer (OR = 60, CI = (16–346), *p =* 4 × 10^−16^). Methylation was significantly more frequent in CRC metastases homing to the ovary and omentum than in those homing to the liver and lung (OR = 6.1, CI = (1.8–22.2), *p =* 0.001). Differentiating local from distant metastatic spread, methylation negatively associated with tumor progression (*p* = 0.031) but positively with depth of invasion (*p* = 0.030). Hypermethylation associated with transcriptional repression in CRC cell lines, and treatment with 5′-AZA-2′-deoxycytidine led to reactivation of mRNA expression. shRNA-mediated silencing of *ADAMTS19* had no effect on the in vitro proliferation rate of CRC cells but significantly diminished their collective migration speed (56 %, *p =* 3.3 × 10^−4^) and potential to migrate in collagen I (64 %, *p =* 4.3 × 10^−10^).

**Conclusions:**

Our results highlight the frequent involvement of *ADAMTS19* epigenetic silencing in CRC and mucinous ovarian cancer. The mechanistic preferences for the target organ of metastatic spread may lead to the development of diagnostic CRC biomarkers. The association with the mucinous phenotype also may have diagnostic applications for ovarian cancer.

**Electronic supplementary material:**

The online version of this article (doi:10.1186/s13148-015-0158-1) contains supplementary material, which is available to authorized users.

## Background

Colorectal cancer (CRC) is the third most common cancer for men and women worldwide with over 1.3 million new cases diagnosed in 2012 [1]. Despite decreasing trends in incidence and mortality in the last decade, CRC still is the third cause of cancer-related deaths accounting for near 10 % of total cancer mortality worldwide [1]. Metastatic disease is the major cause of death in CRC. The metastatic dissemination involves the acquisition by the malignant cell of an abnormal loss of the tridimensional homeostatic tissue organization. Metastatic spread is a complex multistep process that includes several sequential steps: invasion through the extracellular matrix (ECM), migration, epithelial-mesenchymal transition (EMT), angiogenesis, the ability to survive without the contact with other sister cells (“anoikis”), colonization, and resistance to adverse tissue environments [2, 3]. CRC metastasizes most commonly to the liver, the lung, and the peritoneal cavity, and the histological cancer subtypes and tumor location influence the patterns of metastatic spread [3, 4]. Dissemination of metastases in CRC can be roughly classified as via the lymphatic and circulatory systems to distant organs—rectal cancers with tendency for homing to the lung and colon cancers with preferential dissemination to the liver—or via local mesothelial spread of floating cancer cells to the peritoneal surfaces including the omentum and proximal organs [5]. While the first two dissemination ways involve intravasation and extravasation, the third may not, but little is known about the molecular mechanisms that may underlie these different metastatic spread behaviors.

Alterations in the ADAMTS (a disintegrin and metalloproteinase domain with thrombospondin motifs) extracellular matrix metallopeptidases contribute to tumorigenesis and tumor progression [6–8]. The human ADAMTS family encompasses 19 multidomain extracellular matrix metallopeptidases that participate in a wide range of physiological processes, including ECM assembly and degradation, homeostasis, organogenesis, and angiogenesis [7, 9]. The first member of this family, *ADAMTS1*, was cloned in 1997 during a screening of genes selectively expressed on a murine cachexigenic tumor cancer cell line [10]. Multiple other *ADAMTS* genes were later isolated and characterized by several groups [11–21]. ADAMTS enzymes are closely related to the members of the ADAM (a disintegrin and metalloproteinase domain) family of metallopeptidases. However, ADAMTS contain additional thrombospondin type 1 motifs (TSP1) in their sequence. TSP1 motifs are involved in the interaction with glycoconjugates such as heparin and heparan sulfate that are present in the ECM [22, 23]. The physiological substrates of the ADAMTS family members include the propeptides of type I collagen (ADAMTS2 and ADAMTS14), type II collagen (ADAMTS2 and ADAMTS143), aggrecan (ADAMTS1, ADAMTS4, ADAMTS5, ADAMTS9, and ADAMTS12), versican (ADAMTS9), alpha-2-macroglobulin (ADAMTS12), and von Willebrand Factor multimers (ADAMTS13).

According to their physiological functions, ADAMTS proteins have been grouped into anti-angiogenesis (ADAMTS1 and ADAMTS8), aggrecanases (ADAMTS1, ADAMTS4, ADAMTS5, ADAMTS8, ADAMTS9, and ADAMTS15), procollagen N-proteinases (ADAMTS2, ADAMTS3, and ADAMTS14), GON-ADAMTS (ADAMTS9 and ADAMTS20), and the von Willebrand factor cleaving protease (vWCFP, ADAMTS13) [9]. The physiological function and the substrates of ADAMTS6, ADAMTS7, ADAMTS12, and ADAMTS16 to ADAMTS19 remain uncharacterized.

Albeit some experimental data suggests a pro-tumorigenic/metastatic function of ADAMTS proteins, particularly in the case of ADAMTS1, the majority of the results indicate that these proteins have a negative effect on tumor progression [7, 8, 24, 25]. Frequent alterations in the expression of these genes have been found in breast cancer, the majority being downregulated (ADAMTS1, ADAMTS3, ADAMTS5, ADAMTS8, ADAMTS9, ADAMTS10, ADAMTS12, and ADAMTS18) but some being upregulated (ADAMTS4, ADAMTS6, and ADAMTS14) [26]. In addition, mutational inactivation, and more frequently, transcriptional silencing by promoter hypermethylation of ADAMTS genes have been found in different types of cancer [8, 27–32]. We recently reported that promoter hypermethylation of *ADAMTS14* takes place not only in the tumors but also in the non-cancerous colonic mucosa of CRC patients. The methylation of normal colonic mucosa was particularly present in elder African-Americans, suggesting that it is an early event in the carcinogenesis process and a diagnostic marker of a field for cancerization [33]. Nothing is essentially known about the function of *ADAMTS19* or its possible role in cancer.

DNA methylation is an epigenetic mechanism with a profound modulating effect on cellular gene expression patterns. Under normal physiological conditions, it plays a crucial role in establishing cell identity during development and cell proliferation. In many human cancers, however, the normal DNA methylation profile is substantially altered. These alterations comprise both abnormal low levels of genome-wide methylation (DNA hypomethylation) and high levels of methylation in other *loci* (DNA hypermethylation) [34]. Cancer-related DNA hypomethylation mainly occurs in DNA repetitive elements and pericentromeric regions, a phenomenon known to trigger genomic instability [35]. In some cases, DNA hypomethylation has been also found in unique loci leading to transcriptional reactivation [36]. Cancer-related DNA hypermethylation mainly occurs in CpG islands (CGI) associated with gene promoters and is generally accompanied by transcriptional silencing [37]. Since the discovery of the epigenetic inactivation of the tumor suppressor Rb in the late 80s [38], a large number of genes have been found to undergo somatic promoter hypermethylation in human cancer, many of them with tumor suppressor or DNA repair functions [39].

The low or no transcriptional activity of genes with hypermethylated promoter-associated CGI associates with the recruitment of chromatin remodeler complexes that lead to a closed chromatin state [40]. However, after years of intensive research, the primal cause of CGI hypermethylation remains to be defined. The Polycomb (PcGs) and Thritorax (TrxG) group proteins have been suggested to be mechanistically involved in this cancer-specific abnormal hypermethylation, based on the enrichment of PcGs target genes among the genes frequently hypermethylated in cancer [41]. Nevertheless, the reason why some genes may undergo hypermethylation while others do not remains as mysterious today as it was in the over 15-years-old original proposal [42]. The strong association of methylation with mutations in *BRAF* in CRC [43] and isocitrate dehydrogenase 1 gene (*IDH1*) in gliomas [44] provides interesting clues but as indirect pleiotropic effects, rather than direct mechanistic causal relationships.

In this report, we characterized the promoter region of *ADAMTS19* frequently hypermethylated in CRC [45] and extended this observation to other cancers. We correlated methylation with cancer genotype and with clinicopathological parameters, especially the metastatic homing preference. We also studied in vitro the effect of *ADAMTS19* transcriptional silencing in CRC phenotype.

## Results

### Hypermethylation of *ADAMTS19* 5′ CpG island in gastrointestinal primary tumors

**Fig. 1:**
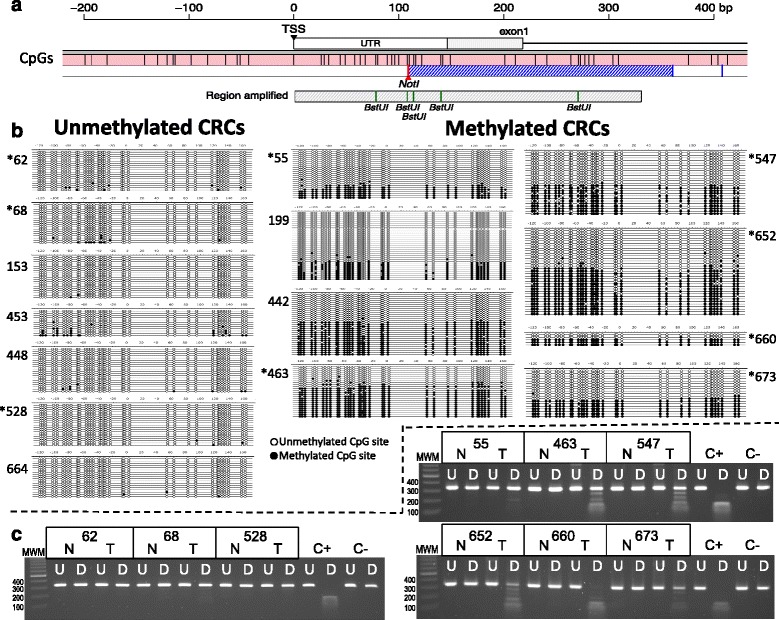
Bisulfite sequencing of exon1 of *ADAMTS19* in 15 CRC cases. **a** Scheme of the 600 bp region surrounding the first exon of *ADAMTS19*. The transcriptional start site (TSS) and 5′ untranslated region (UTR) are indicated. In *pink* is an internal region of the *ADAMTS19* CpG island (CGI), with *vertical bars* indicating every CpG site. The *dashed blue area* indicates the band C-19 initially detected by MS-AFLP. The region amplified for bisulfite sequencing is represented by the *gray rectangle*, with the BstUI sites indicated by *vertical bars*. For a larger view of this region, see Additional file 1: Figure S1. **b** On the left side are cases that were scored as unmethylated by MS-AFLP. On the right are cases that were scored as hypermethylated. Every *horizontal line* represents individual cloned sequences. Every *circle* represents 1 of the 34 CpG sites within the region studied. The scale is relative to the codon +1. In *black and white*, represented are the methylated and unmethylated CpG sites, respectively. Cases with asterisks are included in the representative results of the *Bstu*I COBRA analysis (**c**). *MWM* molecular weight marker (100 bp ladder), *N* normal sample, *T* tumor sample, *C+* positive control (human methylated DNA), *C−* negative control (human non-methylated DNA), *U* undigested, *D* digested. Methylation is indicated by the presence of smaller digestion products

This study spawns from a previous work where we analyzed methylation alterations in colorectal and gastric cancers by methylation-sensitive amplification length polymorphism (MS-AFLP). This is a DNA fingerprinting technique based on the methylation-sensitive cleavage of *Not*I, a restriction endonuclease that contains in its recognition sequence two CpG dinucleotides [45]. A MS-AFLP band named C-19 was the most frequent hypermethylated in both gastric (38/89, 42.7 %) and colorectal (25/73, 34.2 %) cancers [45]. The C-19 band was mapped (see Methods) to the 5′ region of the *ADAMTS19* gene (Fig. 1a).

We confirmed that methylation alterations of the *ADAMTS19* CGI were responsible for the changes in intensity of band C-19 (Additional file 1: Figure S1) [46]. We also analyzed the association between hypermethylation identified by MS-AFLP (which is reflected by weaker intensity of the fingerprint bands) and copy number changes. *ADAMTS19* is located in chromosome 5q, 16.8 Mb telomeric to tumor suppressor *APC*, frequently lost in CRC. Over 50 cases were analyzed for loss of heterozygosity (LOH) and copy number changes in 5q by microallelotyping and array comparative genomic hybridization (CGH) (Additional file 1: Figure S2). There was no association between copy number alterations and the MS-AFLP scoring, showing that band C-19 changes reflected methylation alterations rather than genomic loss of *ADAMTS19*.

We studied the extent of hypermethylation of *ADAMTS19* in 15 CRCs by bisulfite sequencing [47, 48]. The results confirmed that hypermethylation of *ADAMTS19* was exclusive of the tumor tissues and extended throughout the gene promoter region (Fig. 1b). Thus, we used combined bisulfite and restriction analysis (COBRA), a simpler and less expensive method (Fig. 1c), to complete the analysis of 42 colonic adenomas, 210 primary CRCs, 70 metastases (Table 1), and their corresponding normal tissues (for every patient, we analyzed both the tumor and the colonic normal mucosa). *ADAMTS19* was hypermethylated in 48 % of adenomas, 35 % of adenocarcinomas, and 31 % of metastases but never in the 322 normal tissues corresponding to the tumoral samples.

**Table 1:** Demographics and clinical characteristics of the patients and samples analyzed in this study

	Adenomas	Carcinomas	Metastases	Carcinomas vs. Metastases
Number	42	210	70	
Age	*n* = 42	*n* = 210	*n* = 66	
Mean ± SD	64.2 ± 11.4	65.1 ± 14.1	62.5 ± 11.5	*p = 0.12*
Range	30–80	18–93	33–86	
Gender	*n* = 42	*n* = 210	*n* = 66	
Female	13 (31 %)	98 (47 %)	28 (40 %)	
Male	29 (69 %)	112 (53 %)	38 (54 %)	*p = 0.57*
Information not available			4 (6 %)	
Race	*n* = 27	*n* = 161	*n* = 63	
Caucasian	18 (43 %)	125 (60 %)	57 (81 %)	
African-American	9 (21 %)	36 (17 %)	6 (9 %)	*p = 0.035*
Other/not well defined	15 (36 %)	49 (23 %)	7 (10 %)	
Location^a^	*n* = 40	*n* = 210	*n* = 32	
Proximal	27 (64 %)	110 (52 %)	9 (13 %)	
Distal	13 (31 %)	100 (48 %)	23 (33 %)	*p = 0.013*
Information not available	2 (5 %)	0	38 (54 %)	
Stage (Dukes’)		*n* = 210		
A	NA	18 (9 %)	NA	
B	NA	77 (37 %)	NA	
C	NA	59 (28 %)	NA	
D	NA	56 (27 %)	NA	
Grade		*n* = 202	*n* = 32	
Well/moderate	NA	156 (74 %)	32 (46 %)	
Poor	NA	46 (22 %)	2 (3 %)	*p = 0.021*
Information not available	NA	8 (4 %)	36 (51 %)	
Mucinous phenotype		*n* = 210	*n* = 70	
Non-mucinous		151 (72 %)	57 (81 %)	
Mucinous		59 (28 %)	13 (19 %)	*p = 0.15*
MSI	*n* = 41	*n* = 208	*n* = 62	
MSS	40 (95 %)	185 (88 %)	62 (89 %)	
MSI	1 (2 %)	23 (11 %)	0	*p = 0.003*
Information not available	1 (2 %)	2 (1 %)	8 (11 %)	
*TP53*	*n* = 33	*n* = 191	*n* = 42	
WT	28 (67 %)	104 (50 %)	18 (26 %)	
MUT	5 (12 %)	87 (41 %)	24 (34 %)	*p = 0.23*
Information not available	9 (21 %)	19 (9 %)	28 (40 %)	
*KRAS*	*n* = 42	*n* = 195	*n* = 50	
WT	19 (45 %)	117 (56 %)	32 (46 %)	
MUT	23 (55 %)	78 (37 %)	18 (26 %)	*p = 0.63*
Information not available	0	15 (7 %)	20 (29 %)	
*BRAF*	*n* = 12	*n* = 175	*n* = 34	
WT	12 (29 %)	153 (73 %)	32 (46 %)	
MUT	0	22 (10 %)	2 (3 %)	*p = 0.38*
Information not available	30 (71 %)	35 (17 %)	36 (51 %)	

For every parameter, the number of cases with information is indicated (n=)

*NA* not applicable

^a^In the metastases column, location refers to the originating primary lesion when known

*P* values of the comparison between carcinomas and metastases were obtained by Fisher’s test except for Age, where Student’s *t* test was applied

### *ADAMTS19* hypermethylation is independent of hypermethylation of surrounding CpG islands

**Fig. 2:**
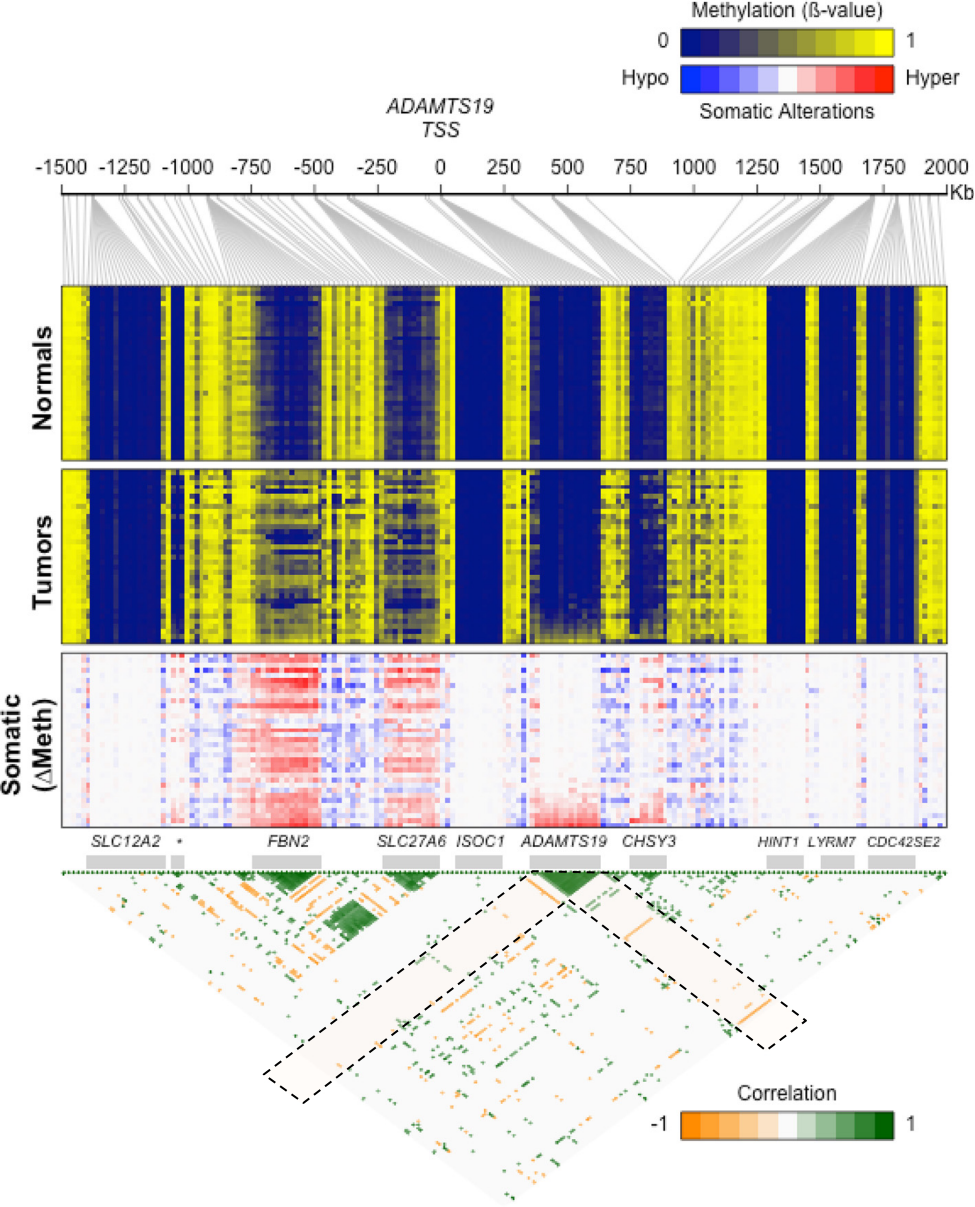
Methylation analysis of the 3.5 Mb region of chromosome 5 surrounding the *ADAMTS19* TSS in colorectal normal samples (*upper heatmap*) and tumors (*middle heatmap*) from 35 CRC patients. *Columns* and *rows* in the heatmaps represent Illumina HM450K probes and tissue samples, respectively. Samples are ordered according to the methylation level of the *ADAMTS19* CGI. The lower heatmap shows the somatic difference in methylation between tumor samples and their matching normal samples. In this region, there are ten CpG islands (*gray bars*), corresponding to the promoters of *SLC12A2*, *FBN2*, *SLC27A6*, *ISOC1*, *ADAMTS19*, *CHSY3*, *HINT1*, *LYRM7*, and *CDC42SE2* genes, as well as an intergenic CGI overlapping with a CTCF binding site (indicated by an *asterisk*). The *lower triangle* shows the correlations between every pair of probes. Only correlations with *r* ^2^ > 0.25 (*p <* 0.01) are shown. The areas corresponding to the correlations with *ADAMTS19* CGI are indicated with *dashed line rectangles*

We studied the methylation of the chr5 123.8–133.8 Mb *ADAMTS19* region in 35 CRCs and their matching normal tissues using Illumina HM450K methylation arrays. The concordance between the COBRA scoring and the Illumina HM450K arrays data was 100 %. Within the 3.5 Mb chromosomal region around *ADAMTS19*, there are ten CGI (Fig. 2). The genes fibrillin 2 (*FBN2*) and *SLC27A6* (a fatty acid transporter) CGIs, located 0.9 and 0.5 Mb upstream of *ADAMTS19*, respectively, were concomitantly hypermethylated. chondroitin sulfate synthase 3 (*CHSY3*) located 0.45 Mb downstream of *ADAMTS19* was also hypermethylated in some tumors. There was no correlation between methylation of *ADAMTS19* CGI and methylation of any of these three genes (Fig. 2), indicating that *ADAMTS19* hypermethylation was an independent event and not a secondary effect of hypermethylation in the neighboring chromosomal region.

### *ADAMTS19* hypermethylation and clinicopathological and molecular parameters in CRC

**Fig. 3:**
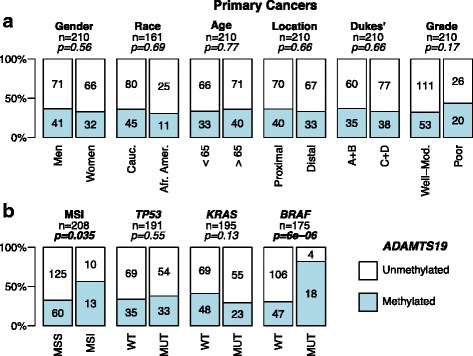
*ADAMTS19* hypermethylation and CRC clinicopathological characteristics (**a**) and tumor genotype (**b**). Tumor stage is indicated using the Dukes’ classification, grouping A and B vs. C and D. *WT* wild type, *MUT* mutated. *P* values were calculated by Fisher’s exact test

*ADAMTS19* methylation in primary CRC did not associate with gender, race, age, or tumor location (Fig. 3a and Additional file 1: Figure S3). We found, however, a positive association with MSI status (odds ratio (OR) = 2.7, confidence interval (CI) = (1–7.3), *p =* 0.035) and with *BRAF* mutations (OR = 10, CI = (3.1–42.9), *p =* 6.3 × 10^−6^) (Fig. 3b). In a multivariate logistic regression analysis including MSI and *BRAF* as factors, the association with *BRAF* mutation retained statistical significance (OR = 10.5, CI = (3.3–43.2), *p =* 2.7 × 10^−4^) while the association with MSI status did not (OR = 0.94, CI = (0.27–2.83), *p =* 0.91).

### *ADAMTS19* hypermethylation profile in gastrointestinal and gynecological cancers

**Fig. 4:**
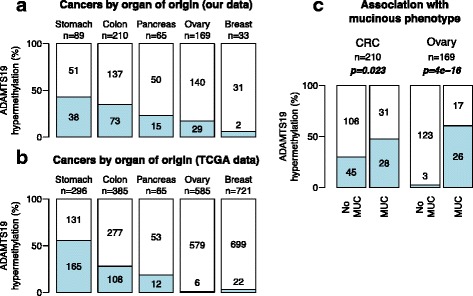
*ADAMTS19* hypermethylation incidence in primary tumors of different origins in tumors from our collection (**a**) and from the TCGA datasets (**b**). In *blue* are *ADAMTS19* hypermethylated cases. A higher incidence is found in gastrointestinal cancers compared to ovary and breast cancers (*p =* 5.2 × 10^−7^, OR = 2.9, CI = (1.9–4.7), Fisher’s exact test). **c** *ADAMTS19* hypermethylation associates with mucinous phenotype in CRC and ovarian cancers (*p* = 0.023 and *p =* 4 × 10^−16^, respectively, Fisher’s exact test). The three ovarian cancer cases with *ADAMTS19* hypermethylation in the no mucinous group were the only three clear-cell adenocarcinomas

To investigate whether *ADAMTS19* hypermethylation was exclusive of gastric and colorectal cancers, we analyzed 356 primary tumor samples from other malignancies. *ADAMTS19* hypermethylation was more frequent in cancers of gastrointestinal origin (stomach, colon, and pancreas) than ovarian and breast cancers (OR = 2.9, CI = (1.9–4.7), *p =* 5.2 × 10^−7^, Fig. 4a).

Analysis of the methylation data from the Cancer Genome Atlas (TCGA) validated our findings (Fig. 4b). In the TCGA datasets *ADAMTS19* was frequently hypermethylated in gastrointestinal cancers including stomach (47.6–53.7 %), esophageal (39.8 %), liver (30.9 %), colorectal (22.2–25.2 %), and pancreatic cancers (13.8 %). In contrast, methylation incidence was much lower in breast (1.5–2.5 %), ovarian (1 %), or prostate (0.5 %) cancers. The TCGA data also confirmed the strong association between *ADAMTS19* hypermethylation and *BRAF* mutations in 220 CRCs (OR = 30.2, CI = (8.3–167), *p =* 4.4 × 10^−11^).

*ADAMTS19* hypermethylation associated with the mucinous phenotype in primary CRCs (OR = 2.1, CI = (1.1–4.2), *p* = 0.023). A much stronger association was observed in ovarian cancers (OR = 60, CI = (16–346), *p =* 3.9 × 10^−16^). None of the serous or endometrioid type tumors, which are the most frequent types of ovarian cancer, exhibited *ADAMTS19* hypermethylation (Fig. 4c). The only other three methylated cases in the non-mucinous subgroup of ovarian cancer were clear-cell tumors.

### *ADAMTS19* methylation associates with local, but not with distant, CRC metastases

**Fig. 5:**
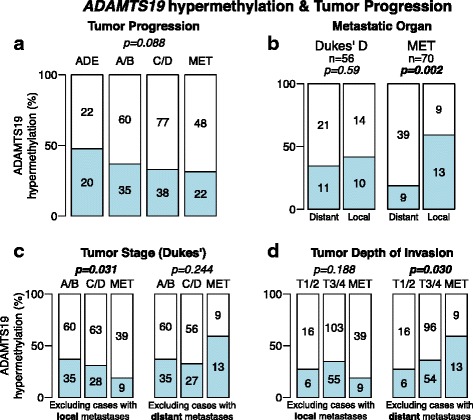
*ADAMTS19* hypermethylation and CRC tumor progression and invasion. **a** Incidence of *ADAMTS19* hypermethylation in adenomas (ADE), primary CRCs (grouping Dukes’ A + B and C + D), and metastases (MET). **b** Incidence of *ADAMTS19* hypermethylation in Dukes’ D primary cancers that metastasized to distant (mostly the liver or lung) or to local (mostly the ovary or omentum) organs and in metastases (MET). **c** Incidence of *ADAMTS19* methylation vs CRC progression in primary cancers (Dukes’ scale, grouping A/B and C/D). **d** Incidence of *ADAMTS19* methylation and depth of invasion in primary cancers (TNM scale, grouping T1/2 and T3/4). *P* values were calculated by Fisher’s exact test (**b**) and by Cochran-Armitage test for trends (**a**, **c**, and **d**)

*ADAMTS19* hypermethylation was frequent in adenomas and showed a trend for decreasing frequency during tumor progression (Fig. 5a). *ADAMTS19* methylation was significantly higher in local metastases to the omentum and ovary than to distant organs, the liver and lung (OR = 6.1, CI = (1.8–22.2), *p =* 0.0017, Fig. 5b). By differentiating between distal and local metastases, inverse trends became significant. Thus, methylation decreased from non-metastatic CRCs (Dukes’ A + B) to metastatic CRCs (Dukes’ C + D) to metastases to the liver and lung (*p* = 0.031, Cochran-Armitage test) (Fig. 5c). Inversely, association with the depth of invasion revealed an increased incidence of methylation in more invasive tumors and their corresponding local metastases (*p* = 0.030, Cochran-Armitage test) (Fig. 5d).

### Methylation associates with gene silencing and demethylation restores *ADAMTS19* expression

Genomic DNA from Colo205, DLD1, HCT8, HCT15, HCT116, HT29, LoVo, LS180, SW48, and SW480 CRC cell lines was treated with bisulfite, polymerase chain reaction (PCR) amplified, and cloned. Five plasmid clones from each cell line were isolated and sequenced. The results revealed different methylation patterns with some of the cell lines heavily methylated, while others were essentially unmethylated (Additional file 1: Figure S4). The expression level of *ADAMTS19* in these cell lines was analyzed by quantitative reverse transcription polymerase chain reaction (RT-PCR). Cell lines with full methylation of *ADAMTS19* CGI did not exhibit detectable levels of expression. Some of the cell lines with intermediate levels of methylation expressed the gene at very low level, but the highest level of expression corresponded to the fully demethylated cell lines DLD1/HCT15 and SW480 (Additional file 1: Figure S4). We studied the association between methylation and transcriptional levels by treating these cell lines with the demethylating agent 5-AZA-2-deoxycytosine (AdC). After 48 h of treatment, *ADAMTS19* expression was analyzed by RT-PCR. The results confirmed that pharmacological demethylation with AdC restored expression in the four fully methylated cell lines (HT29, SW48, Colo205, and HCT116) (Additional file 1: Figure S4).

### *ADAMTS19* downregulation reduces the migration capabilities of CRC cells

To investigate the phenotypic effect of *ADAMTS19* silencing, we performed knockdown experiments with interference RNA (shRNA) in DLD1 and SW480 cell lines, having no methylation and exhibiting the highest levels of expression (Additional file 1: Figure S4b). We designed three different specific shRNAs targeting exons 3, 13, and 22 (shA19e3, shA19e13, and shA19e22, respectively). These shRNAs were transfected into SW480 and DLD1 separately and in different combinations. As negative controls, cells were transfected with vectors containing shRNA targeting luciferase (shLuc) or GFP (shGFP1 and shGFP2) genes, both absent in these cells. Transfected cells were selected by culture with puromycin. *ADAMTS19* transcriptional levels were analyzed by RT-PCR to determine the efficiency of these shRNAs in stably transfected cells. The most efficient silencing was achieved with shA19e22, which downregulated the levels of expression of *ADAMTS19* to less than 30 % of untreated levels in SW480 (Additional file 1: Figure S5). Similar results were obtained in DLD1. To investigate whether some subclones achieved even stronger downregulation, 10 subclones of the shA19e22 transfected SW480 cells were isolated and individually evaluated for *ADAMTS19* expression. These subclones exhibited little deviation from the silencing level measured in the cell pool (Additional file 1: Figure S5).

**Fig. 6:**
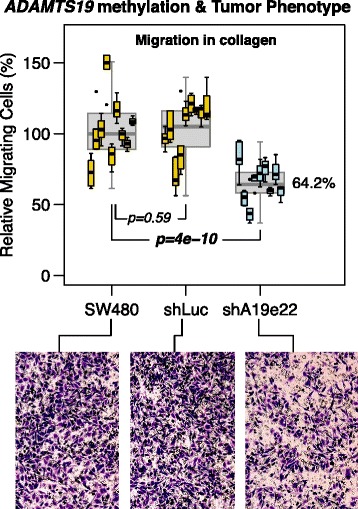
Silencing of *ADAMTS19* with shA19e22 reduces the migration capabilities of SW480 cells. Transwell migration assay using a matrix of collagen I. Experiments were performed in three independent replicates, using three wells per cell line in each replicate, and examining three to six microscope fields per well. No statistically significant difference was detected between replicates. In *gray* are boxplots combining all the values per cell line. In *orange* are boxplots showing the values of individual wells in controls (SW480 and SW480 transfected with shLuc) and in *blue* the *ADAMTS19*-silenced cell line SW480 transfected with shA19e22. A 35.8 % reduction of median migratory capabilities was observed in SW480 cells transfected with shA19e22. *P* values were calculated by Tukey’s honest significance method on a nested ANOVA model

*ADAMTS19* silencing did not affect the in vitro growth rate or anchorage-free growth capabilities (Additional file 1: Figure S6) of *ADAMTS19*-downregulated SW480 cells. We also studied changes in invasion potential in vitro by Matrigel-coated Transwell assays. However, the only parental cell lines with high expression of ADAMTS19 (DLD1/HCT15 and SW480) exhibited very low capability to migrate through the Matrigel layer (averaging 1 or 2 cells per view field), yielding no statistically significant observable difference between the cells with and without knockdown of gene expression (not shown). However, we found a significant reduction in the migratory capabilities of SW480 cells upon *ADAMTS19* downregulation, measured by two complementary methods, i.e., wound healing (Additional file 1: Figure S7) and collagen I coated Transwell assays (Fig. 6).

## Discussion

We were intrigued by the observation that *ADAMTS19* hypermethylation was the most common epigenetic alteration observed in gastric and colorectal cancers among the many loci analyzed by unbiased MS-AFLP fingerprinting. The rationale to investigate this finding in more depth seemed justified because of the established role of ADAMTS proteins in tumorigenesis, and at the same time, the unexplored nature of the ADAMTS19 in the process. Once the fingerprinting observation was validated by direct bisulfite sequencing and other complementary epigenomic techniques, we explored in a descriptive study the involvement of this somatic epigenetic alteration in several malignancies. The results showed a specific association with gastrointestinal cancers that was corroborated by in silico analysis of the public TCGA data. Methylation also associated with silencing and demethylation in vitro by azacytidine restored expression.

We then correlated the methylation alterations with clinicopathological and genetic parameters in CRC. No significant associations were found with any of the demographic, pathological, or genetic data analyzed except with the MSI status and mutations in *BRAF* (most of them MSI cancers, Fig. 2). This is consistent with previous observations showing that *BRAF*-mutant CRCs exhibit a higher frequency of somatic CGI hypermethylation.

When analyzed in relation to metastatic spread, methylation showed a much lower association with metastases homing to distant (i.e., the lung and liver) organs in comparison with those metastases to local organs such as the ovary and omentum. Finally, in an effort to find a connection genotype-phenotype, we studied several cellular phenotypes related to cell growth and migration. The results overall are not conclusive, and the putative role in CRC pathogenesis of the epigenetic silencing of *ADAMTS19* remains to be established. However, we have several clues that point to an active contributory role in CRC tumor progression.

First, the tumor-specific somatic hypermethylation does not appear to be a random and general epiphenomenon because it is restricted to gastrointestinal malignancies, as it is essentially absent in ovarian and breast cancers. Moreover, methylation associates with the manifestation of the mucinous phenotype in CRC and especially in ovarian cancer. The striking association between *ADAMTS19* methylation and the mucinous phenotype in ovarian cancers, a subtype of cancer of difficult diagnosis due to its resemblance to secondary lesions of colorectal origin, opens a number of avenues for further investigation with potential diagnostic applications.

Second, methylation is not consequence of a local epigenetic disturbance in the genome driven by a nearby “culprit” gene. This conclusion is supported by the discontinuous map of hypermethylation in the *ADAMTS19* region and the absence of association between methylation of *ADAMTS19* and any of the adjacent genes, some of which are indeed methylated in CRC even with higher frequency (Fig. 2). In particular, *FBN2*, is an obvious candidate for the targeted hypermethylation of this chromosomal region because mutations in the gene have been linked to Marfan-like syndromes [49] and because hypermethylation has been reported as a biomarker in CRC and other cancers [50–52]. However, notwithstanding a putative independent role of *FBN2* in CRC, we conclude that *FBN2* is not the main target for inactivation because of the following considerations: Methylation of this gene is not specific for tumor cells as it shows some weaker methylation in normal tissue, which is not the case for *ADAMTS19* (Fig. 2). Moreover, *FBN2* is not included in the microdeletion of this chromosomal region present in one of the CRCs analyzed by array CGH that targets only the *ADAMTS19* gene and the very few adjacent genes. The result is conclusive because a polymorphic CA repeat located outside the *FBN2* gene retained heterozygosity in this tumor, showing that *FBN2 was* not affected by the microdeletion (Additional file 1: Figure S2). This microdeletion also adds evidence for the existence of a selective pressure for the loss of *ADAMTS19* in the early stages of CRC.

In contrast with the absence of association between hypermethylation of *FBN2* and *ADAMTS19*, there is a coordinated hypermethylation of this metallopeptidase and other members of the *ADAMTS* gene family that extends across the genome (data not shown). The mechanism underlying this intriguing trans-acting positive correlation in *ADAMTS* methylation is inexplicable at the moment and deserves further analysis. Nevertheless, it also serves as a comparative control over the lack of correlation with the genes adjacent to *ADAMTS19*, showing that the gene is a target in itself for somatic hypermethylation in a subset of CRC.

Third, the most direct evidence for a functional role of *ADAMTS19* hypermethylation is the observed phenotypic changes after downregulation of its expression. This proves that silencing is not inconsequential. However, there is no sufficient data to provide a clear picture of the actual role of ADAMTS19 and its epigenetic alteration in CRC tumorigenesis because of the complex features so far emerging of its involvement. Silencing and downregulation accompanied by inhibition of migration is not easily reconciled with a functional role in tumor progression. However, the asymmetries observed in the incidence of methylation during tumor progression and metastatic spread may indeed provide some potential explanations. A positive selection for cells with a methylated and silenced gene during the early stages of CRC development may be followed by a reverse selection in more advanced stages of tumor invasion and progression. This also is to be understood in the context of the different behavior of the epigenetic alterations depending on the different parameters ruling tumor progression: There appears to be a negative association with stage of progression but a positive association with the depth of invasion (Fig. 5). Similarly, there is a decreased of incidence in methylation in distant hepatic and lung metastases but an increased incidence of local metastases such as the ovary and omentum (Fig. 5).

*ADAMTS19* hypermethylation frequency was higher in adenomas (47.6 %) than in carcinomas (34.6 %) or metastases (30.9 %). Considering MSS tumors only, the results were essentially the same, 50 % in adenomas, 32.4 % in adenocarcinomas, and 29.5 % in metastases, revealing an asymmetry between premalignant and malignant tumors (OR = 0.47, CI = (0.22–0.97), *p* = 0.031). This suggests that *ADAMTS19* hypermethylation could be detrimental for tumor progression, i.e., adenomas with *ADAMTS19* hypermethylation would be less likely to become malignant or carcinomas less metastatic. However, our data is the endpoint analysis of different samples and does not necessarily represent an accurate time-course of the adenoma-carcinoma-metastasis progression. The cases with primary-metastases from the same CRC patients were too few to reach meaningful conclusions in this regard, but to increase the sample size of these cases is an obvious course of action in the future.

The main difficulty in proposing a coherent model for the role of *ADAMTS19* hypermethylation in CRC resides in the generally accepted irreversibility of aberrant hypermethylation. Why a gene that undergoes hypermethylation early on during tumorigenesis would have a drop in methylation incidence later on the process is not altogether clear. For the reasons discussed before, the first “passenger” hypothesis to explain methylation as inconsequential seems unlikely. The simplest explanation for a functional role of hypermethylation is that the primary tumor may be heterogeneous for methylation status, with the cells that eventually disseminate and colonize the liver, for instance, coexisting unmethylated in the primary tumor with other methylated sister cells. In support of this hypothesis, some of the clones from *ADAMTS19* methylation-positive tumor samples analyzed by bisulfite sequencing (Fig. 1b) were essentially unmethylated, resembling the methylation pattern of the normal tissue. The most likely explanation is that *ADAMTS19* methylation is heterogeneous in the cell population. In addition to contamination with normal cells (obligated in primary tumor samples), non-clonal methylation may account for this heterogeneity.

Our in vitro studies indicate that *ADAMTS19* downregulation reduces the motility of cancer cells (Fig. 6 and Additional file 1: Figure S7), suggesting that *ADAMTS19* hypermethylated cells could be less capable to escape the tumor mass and migrate to distant organs through the vascular system. This effect was observed despite the fact that shRNA-induced downregulation did not completely silence *ADAMTS19* transcription. The analysis of mRNA expression in cell lines (Additional file 1: Figure S4) suggests that promoter hypermethylation has a stronger downregulation effect. Therefore, it is possible that in vivo *ADAMTS19* hypermethylation exerts a stronger negative effect on the migration capabilities of tumor cells. This hypothesis predicts that some of the clonally methylated tumors will generate methylated metastases, preferentially to local organs (the ovary or omentum), while other tumors non-clonal for methylation will generate unmethylated metastases preferentially to distant organs (the liver or lung). While metastatic spread to distant organs involves intra- and extravasation, the dissemination into the peritoneal cavity may occur by direct spread of floating tumor cells once the primary tumor invades and penetrates through the colon wall. Inhibition of migration would not hamper the metastatic dissemination in this context. This is most likely an over-simplifying hypothesis but at least provides a working model for the differences in metastatic homing by the cells with and without *ADAMTS* methylation.

## Conclusions

In summary, our results demonstrate that the promoter of *ADAMTS19* is targeted by hypermethylation in a significant proportion of gastrointestinal cancers, particularly in *BRAF*-mutant cancers, and that this hypermethylation associates with transcriptional downregulation and reduces the in vitro migration capabilities of CRC cells. The link between methylation of this gene and altered in vivo migration and invasion capabilities of metastatic cells remains to be established. A more detailed study with animal model systems for metastasis of CRC seems an obvious approach. All together, our findings reinforce the emerging role of extracellular matrix homeostasis disruption as a relevant event in cancer progression in general and CRC in particular [8, 53, 54].

## Methods

### Cell lines and human tissues

Freshly frozen human cancers and normal matching tissues (from each one of the patients) were obtained from the Cooperative Human Tissue Network [55]. Colon cancer cell lines Colo205, DLD1, HCT8, HCT15, HCT116, HT29, LoVo, LS180, SW48, and SW480 were obtained from the American Type Culture Collection, Rockville, MD, USA. Cell lines were authenticated by STR profiling using Identifiler Plus PCR Amplification kit (Life Technologies). DLD1 and HCT15 cell lines are in fact the same, as they were derived from the same tumor, although due to their mutator phenotype they harbor several genotypic differences [56]. Sanford-Burnham Institutional Review Board approved the research protocol, which was in compliance with national legislation and performed according to the ethical guidelines of the Declaration of Helsinki [57].

### Cell culture conditions

Cells were cultured in DMEM-F12 (Life Technologies) supplemented with fetal bovine serum 10 % (*v/v*), antibiotics, and antimycotics on 100 mm culture dishes in a 37 °C incubator with 5 % CO_2_. Unless otherwise indicated, cells were grown until reaching 80–90 % confluency before collection. When needed, 5-aza-2′-doexycytidine (5AdC) was added to the culture media at a final concentration of 1 μM.

### DNA methylation analyses

MS-AFLP was performed as previously described, using primer NotI + G in three separate combinations with primers MseI + CA, MseI + CG, and MseI + C [45, 58]. We used a *Sac*II-based quantitative analysis for methylation, a method similar to MethylScreen [46], with some modifications. Briefly, genomic DNA was first sheared by digestion with *Eco*RI at 37 °C during 2 h. Then the sample was divided into two aliquots. One of them was treated with the methylation-sensitive enzyme *Sac*II at 37 °C for 4 h, while the other was subjected to the same incubation but in the absence of restriction enzyme. Then, the percentage of methylated molecules was estimated as the proportion of DNA molecules resistant to digestion in the *Sac*II-treated aliquot, evaluated by quantitative PCR in a LightCycler 480 System (Roche), with primers P28 and P29 (see Additional file 1: Table S1), and using the *Sac*II-untreated aliquot as reference. For bisulfite sequencing [47, 48] and combined bisulfite and restriction analysis (COBRA) [59], 1 μg of genomic DNA was treated with bisulfite (EZ-methylation kit, Zymo Research). Human methylated and non-methylated DNA standards (Zymo Research) were used as controls. After bisulfite treatment, the promoter region of *ADAMTS19* was amplified by a two-step nested PCR. Conditions for the first amplification step were 100 ng of bisulfite-treated DNA as template, primers P44 and P46 at a final concentration of 0.4 μM each, dNTPs at 0.125 μM each, Q-Solution 0.5X, and one unit of Hotstart DNA Polymerase (Qiagen) in a total volume of 20 μL. The PCR program consisted of 1 cycle at 95 °C for 5 min to activate the enzyme, followed by 35 cycles of denaturation at 95 °C for 5 s, annealing at 55 °C for 30 s, and extension at 72 °C for 60 s, ending at 72 °C for 5 min to complete extension. The product of the first amplification was diluted 1:20 (*v/v*) in TE 0.1X (Tris · HCl 1 mM, EDTA 0.1 mM, pH 8.0). One microliter of the dilution was used as template for a second reaction using identical conditions but with primers P16 and P45. Shorter PCR programs, using as low as 20 + 25 cycles yielded essentially identical results. The nested PCR generated a single amplicon of 331 bp. For COBRA analysis, 5 μL of PCR product were treated for 1 h with *Bst*UI (New England Biolabs) at 60 °C or with its isoschizomer *Bsh*1236I (Thermo Scientific) at 37 °C, both recognizing the 5′-CGCG-3′ sequence. In parallel, 5 μL of the PCR product were subjected to incubation in the same conditions but in the absence of restriction enzyme. After digestion, samples were resolved by electrophoresis in 2 % (*w/v*) agarose gels or, in some cases that required higher sensitivity, in 8 % acrylamide/bisacrylamide (29:1) vertical gels. After electrophoresis, gels were stained with ethidium bromide and visualized in a GelDoc XR system (Biorad). Methylation was determined by the presence of digestion products in the restriction enzyme-containing reaction that indicate the presence of originally methylated CGCG sites resilient to the bisulfite conversion. For bisulfite sequencing, 1 μL of PCR product was cloned into pCR2.1-TOPO vector (Invitrogen) following manufacturer’s instructions and transformed into *E. coli* TOP10 competent cells. Transformed cells were selected onto LB plates containing Ampicillin (50 μg/mL) and X-Gal (40 μg/mL). Ten to 20 white colonies were selected for plasmid preparation (QIAprep miniprep kit, Qiagen, CA). The plasmid inserts were sequenced using primers M13-forward and M13-reversal (Qiagen). Array-based methylation analyses were performed on Infinium HumanMethylation450 BeadChip arrays and scanned in a HiScanSQ system (Illumina, CA), following the manufacturer’s instructions. Bioinformatic analysis was performed using RnBeads package [60].

### Gene expression analyses

To analyze *ADAMTS19* mRNA levels, total RNA was extracted using TRIzol Reagent (Invitrogen, Life Technologies) and used as template to synthesize cDNA using Superscript-II reverse transcriptase (Invitrogen, Life Technologies) with random hexamers for priming. We designed two primers that anneal in exon 20 (primer PB176) and exon 21 (primer PB177), generating a 242 bp amplicon. The amplification was quantified in real time using SYBR-Green Master Mix in a Lightcycler LC480-II System (Roche, CA). After 40 cycles, the specificity of the amplification was verified by melting curve analysis, and the amplicon size was subsequently confirmed by electrophoresis in 2 % (*w/v*) agarose gels. All reactions were performed in duplicate. Expression levels were calculated using the 2^−∆∆Ct^ method combining both *GAPDH* and *TPT1* as normalization genes. In all reactions, efficiency was very close to 2 within the range of concentrations assayed.

### Microallelotyping and array CGH analyses

Copy number alterations were analyzed by microallelotyping using polymorphic dinucleotide microsatellite markers D5S642 and D5S2057, located 0.6 Mb centromeric and 1.8 Mb telomeric of *ADAMTS19*. In some cases where both markers were in homozygosis, we also analyzed D5S2098, located 5 Mb upstream of *ADAMTS19*. Primer sequences to amplify these markers were obtained from the Ensembl website [61]. PCR amplification was performed in presence of α-^32^P-dCTP and resolved in vertical electrophoresis acrylamide-bisacrylamide gels. After electrophoresis, gels were dried and exposed to X-ray films. Loss of heterozygosity was assessed in heterozygous cases by the relative change in intensity in one of the bands when comparing the normal and tumor sample. aCGH was performed using Agilent 44K arrays, following the manufacturer’s protocol. Copy number alterations were analyzed using Agilent Genomic Workbench, with ADM-2 algorithm, threshold of 6, and Fuzzy Zero correction. Only alterations with a minimum of three consecutive probes were considered valid.

### shRNA design and transfection

Three pairs of oligos coding for the shRNAs and targeting *ADAMTS19* exons 3 (Exon3-F and Exon13-R), 13 (Exon13-F and Exon13-R), and 22 (Exon22-F and Exon22-R) were designed using the web server from Life Technologies [62] (Additional file 1: Table S1). Two hundred picomole of every primer pair was annealed by incubation at 95 °C for 4 min and a stepwise cooling of 5 °C every 4 min down to 40 °C. The annealed oligos were cloned into pSUPER (RNAi system, oligoengine) following the manufacturer’s indications. After cloning, the shRNA-containing plasmids were purified and transfected into the target cells using GeneJuice Transfection Reagent (Novagen). Stable transfected cells were selected by culturing in the appropriate culture medium supplemented with 5 μg/mL puromycin.

### In vitro proliferation assay

Cell proliferation was measured using a colorimetric test based on the capability of metabolic active cells to cleavage the yellow tetrazolium salt (XTT) to form a soluble orange formazan dye tetrazolium salt (Cell Proliferation Kit II XTT, Roche). Briefly, the cells were plated in 96-well microtiter plates at a density of 2 × 10^4^ cells/well in Dulbecco’s modified Eagle’s medium (DMEM) medium with 10 % fetal bovine serum (FBS) and cultured at 37 °C for 0, 12, 24, 48, and 72 h in a humidified atmosphere of 5 % CO_2_. Then, 50 μl of XTT was added to each well and incubated for 4 h at 37 °C in the presence of 5 % CO_2_. After the incubation with XTT, the optical density was measured at 492 and 690 nm using a plate reader. The amount of metabolic active cells was estimated by subtracting the OD_690nm_ value to the OD_492nm_ value, as indicated by the kit manufacturer.

### In vitro anchorage-free growth assay

Cells were suspended in growth media containing 0.3 % (*w/v*) agarose at 5 × 10^3^ cells/ml and layered over 1 % (*w/v*) agar in growth media in 35 mm plates. The agar was allowed to solidify at room temperature for 20 min before incubating the cells at 37 °C and 5 % CO_2_. After 21 days, colonies were stained with 0.5 % (*w/v*) Crystal Violet (0.5 %) in 10 % (*v/v*) ethanol, then photographed, and counted. All the samples were assayed in triplicate, and in each replica, a minimum of three fields were counted and averaged.

### In vitro migration assay

Cell migration was assayed by two complementary methods: scratch/wound healing assays and Transwell plates (8 μm pore size) (Millicell, Millipore). For the scratch/wound healing assays, cells were cultured until reaching confluency and then four different scratches were done using a P200 micropipette tip. The width (in µm) of the scratches was measured at different positions using an automatized-capture Leica DMI 6000 B microscope. After 24 h of incubation, the width was measured again at the same coordinates that were previously stored in the microscope managing software. The collective migration speed was estimated dividing the difference in the scratch width by two, and then by 24h (see equation in figure S7). Clumps or colonies of cells inside the scratch but disconnected from the borders of the scratch were ignored. The experiments were performed in duplicate. For the Transwell plate assays, the undersurface of the membrane was coated at 4 °C overnight with 40 μg/mL of Collagen I (BD Bioscience, cat N. 354236) diluted in PBS and then blocked with 2 % (*w/v*) BSA at room temperature for 2 h. The upper compartment was seeded with 2 × 10^5^ o/n starved transfected cells per well in 200 μL of serum-free DMEM + 0.5 % BSA. DMEM + FBS (10 %) was added in the lower chamber. Cells were allowed to migrate through the membrane for 21 h. Cells capable of migrating through the membrane were stained with 0.5 % (*w/v*) Crystal violet (Sigma) in 10 % (*v/v*) ethanol. Each experiment used quadruplicate wells and, within each well, counting was performed in six randomly selected microscopic high-power fields (100×).

### In vitro invasion assay

Cells stably transfected with the shRNAs were starved overnight in serum-free DMEM and then loaded into Matrigel invasion chambers (24 wells, BD Biocoat Matrigel invasion chamber, BD Biosciences) at a density of 2.5 × 10^4^ cells/well, according to the manufacturer’s instructions. Cells were allowed to invade for 24 h, and then invasive cells were fixed and stained with 0.5 % (*w/v*) Crystal violet in 10 % (*v/v*) ethanol and counted using an inverted microscope (Leica, McBain Instruments, Chatsworth, CA). Each experiment used quadruplicate wells, and within each well, counting was performed in six randomly selected microscopic high-power fields (100×).

### Analysis of the TCGA data

Methylation status of *ADAMST19* CGI was downloaded from the methHC webserver [63]. Methylation data of the TCGA ovarian cancer dataset is not included in methHC because most cases have been analyzed with the HM27K platform. Hence, this dataset was directly downloaded from the TCGA. COAD (colon) and READ (rectum) datasets were combined into a single dataset, representing colorectal carcinomas (CRC).

### Statistical analysis

Statistical analyses were performed using the R statistical environment [64]. Association between two categorical variables was analyzed by Fisher’s exact test (for 2 × 2 contingency tables) or chi-square test (for larger contingency tables). Normality of continuous variables was assessed using the Shapiro-Wilk test. Comparisons between two groups were performed with the Student’s *t* test for variables following a normal distribution or with the non-parametric Mann-Whitney-Wilcoxon test for variables that do not follow a normal distribution. When more than two groups were analyzed, we applied ANOVA or rANOVA analyses followed by Tukey’s honest significant difference method. Trend analysis of categorical data was performed using the Cochran-Armitage test. The level of statistical significance was set at *p <* 0.01, unless otherwise specified. Holm’s multi-hypothesis testing correction was applied when appropriate [65].

## Additional file

Additional file 1: Figures S1–7 and Table S1. Validation of the methylation alterations in *ADAMTS19*, array CGH analysis of Chr5, association of *ADAMTS19* with clinicopathological parameters, methylation and expression of *ADAMTS19* in CRC cell lines, silencing of *ADAMTS19* expression using shRNA constructs, effect of *ADAMTS19*-silencing on growth rate and anchorage free growth, effect of *ADAMTS19*-silencing on collective cell migration speed, and sequence of primers used in this study.

## Abbreviations

CGH: comparative genomic hybridization
CGI: CpG island
CI: 95 % confidence interval
COBRA: combined bisulfite and restriction analysis
CRC: colorectal cancer
LOH: loss of heterozygosity
MS-AFLP: methylation-sensitive amplified fragment length polymorphism
OR: odds ratio
PCR: polymerase chain reaction
TCGA: the Cancer Genome Atlas
TSS: transcriptional start site
