# Supplementary material for: Epigenetic inactivation of the extracellular matrix metallopeptidase ADAMTS19 gene and the metastatic spread in colorectal cancer
Source: Clin Epigenetics. 2015 Dec 2;7:124. doi: 10.1186/s13148-015-0158-1 (PMC4667455; doi:10.1186/s13148-015-0158-1)
Supplement: Additional file 1: Figures S1–7 and Table S1. — Validation of the methylation alterations in ADAMTS19, array CGH analysis of Chr5, association of ADAMTS19 with clinicopathological parameters, methylation and expression of ADAMTS19 in CRC cell lines, silencing of ADAMTS19 expression using shRNA constructs, effect of ADAMTS19-silencing on growth rate and anchorage free growth, effect of ADAMTS19-silencing on collective cell migration speed, and sequence of primers used in this study. [file 13148_2015_158_MOESM1_ESM.pdf]

Epigenetic inactivation of the extracellular matrix metallopeptidase *ADAMTS19* gene, and the metastatic spread in colorectal cancer.

SUPPLEMENTARY INFORMATION

Table of contents

	Page
Figure S1. Validation of the methylation alterations in *ADAMTS19*	2
Figure S2. Array CGH analysis of Chr5	4
Figure S3. Association of *ADAMTS19* with clinicopathological parameters	5
Figure S4. Methylation and expression of *ADAMTS19* in CRC cell lines	6
Figure S5. Silencing of *ADAMTS19* expression using shRNA constructs	7
Figure S6. Effect of *ADAMTS19*-silencing on growth rate and anchorage free growth	8
Figure S7. Effect of *ADAMTS19*-silencing on collective cell migration speed	9
Table S1. Sequence of primers used in this study	10

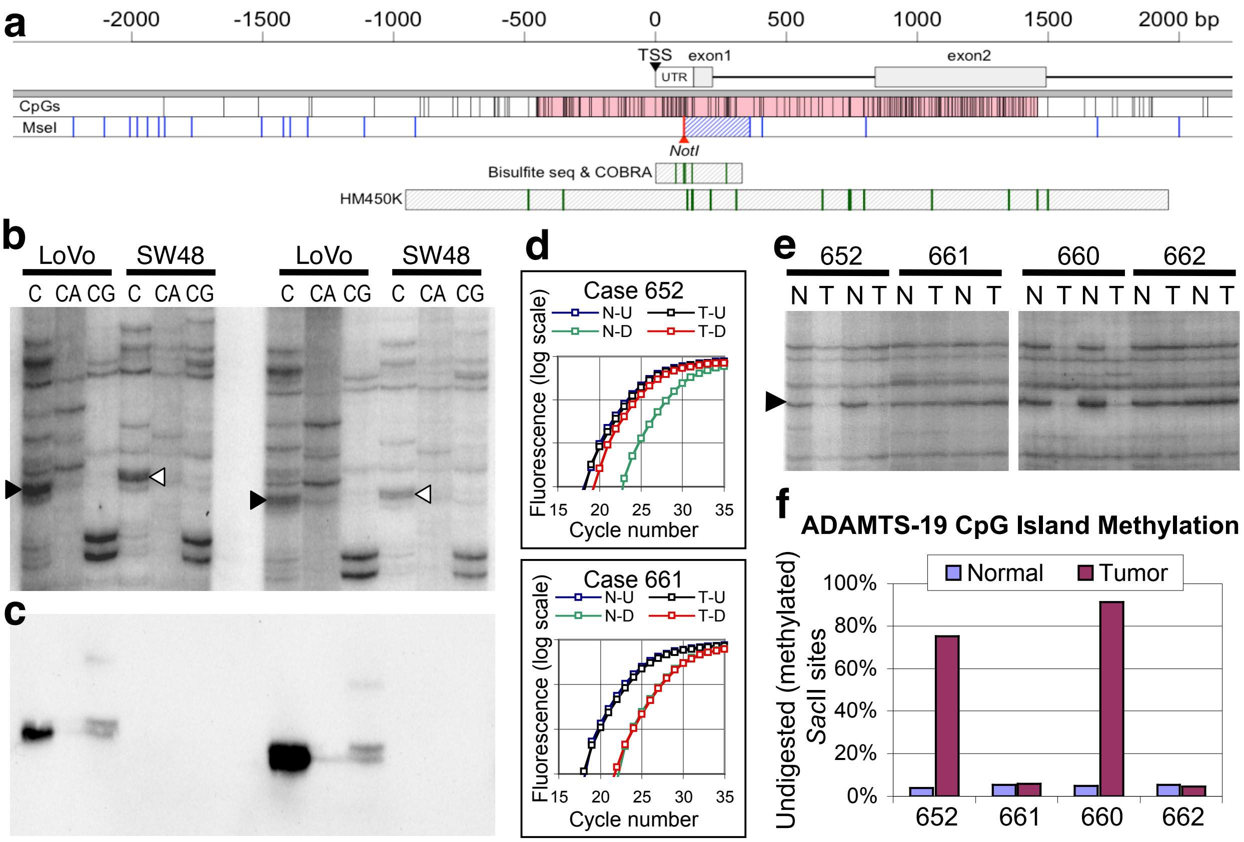

Figure S1. a) Diagram of the *ADAMTS19* 5' region. Coordinates are relative to the transcriptional start site (TSS). Below, it is indicated the position of all the CpG sites (CpGs, black bars) and the annotated CpG island (in pink) in the sequence, and the position of the *Mse*I sites (blue bars) and the *Not*I site (red bar) detected by MS-AFLP. The dashed blue rectangle indicates the *NotI-MseI* band C-19. The dashed grey rectangles represent the region analyzed by bisulfite sequencing and COBRA (green bars: BstUI sites), and the region selected for analysis with Illumina HM450K arrays (green bars: probes within this region). b) Southern blot analysis on a MS-AFLP gel with DNA from LoVo and SW48 cell lines, that according to previous MS-AFLP gels were unmethylated and methylated, respectively. Autoradiography of an MS-AFLP gel on DNA samples from LoVo and SW48 cell lines (in duplicate) using primer NotI+G in combination with MseI-C (C), MseI-CA (CA) or MseI-CG (CG). Band C-19 is indicated by the black triangles. White triangles indicate a different band of undetermined origin that migrates slightly higher in the SW48 samples. c) Autoradiography of the Southern blot performed on the gel shown in the upper panel using a ^{32}P-labeled a 120bp PCR amplicon corresponding to the internal sequence of band C-19 as hybridization probe. The hybridization probe recognizes band C-19 in the MseI-C fingerprint of LoVo (unmethylated cell line) and, in lower extend, some bands in the MseI-CA and MseI-CG fingerprints from the same cell line, but it does not generate a detectable signal in the SW48 (methylated cell line) fingerprints. d) Representative results of the MethylScreen qPCR amplification of normal (N) and tumor (T) samples from patient 652 (upper panel, hypermethylated) and patient 661 (lower panel, not hypermethylated). Amplification was performed using

primers P28 and P29 on normal and tumor DNA samples digested with SacII (N-D and T-D, respectively), which overlaps with the *Not*I site depicted in panel a. Normalization was performed by parallel amplification of the undigested samples (N-U and T-U). Methylation protects the DNA from digestion, resulting in a smaller ∆Ct between the digested and undigested samples e) Region of MS-AFLP gels showing the intensity changes in band C-19 (black triangles) that reflect methylation changes in *ADAMTS19* in two unmethylated cases (661 and 662) and two methylated cases (652 and 660). Hypermethylation causes a decrease in the intensity of band C-19. f) Quantification of the percentage of molecules resistant to digestion (i.e. methylated) in the 4 samples showed in panel e. There is a perfect match between the results obtained by MS-AFLP and MethylScreen.

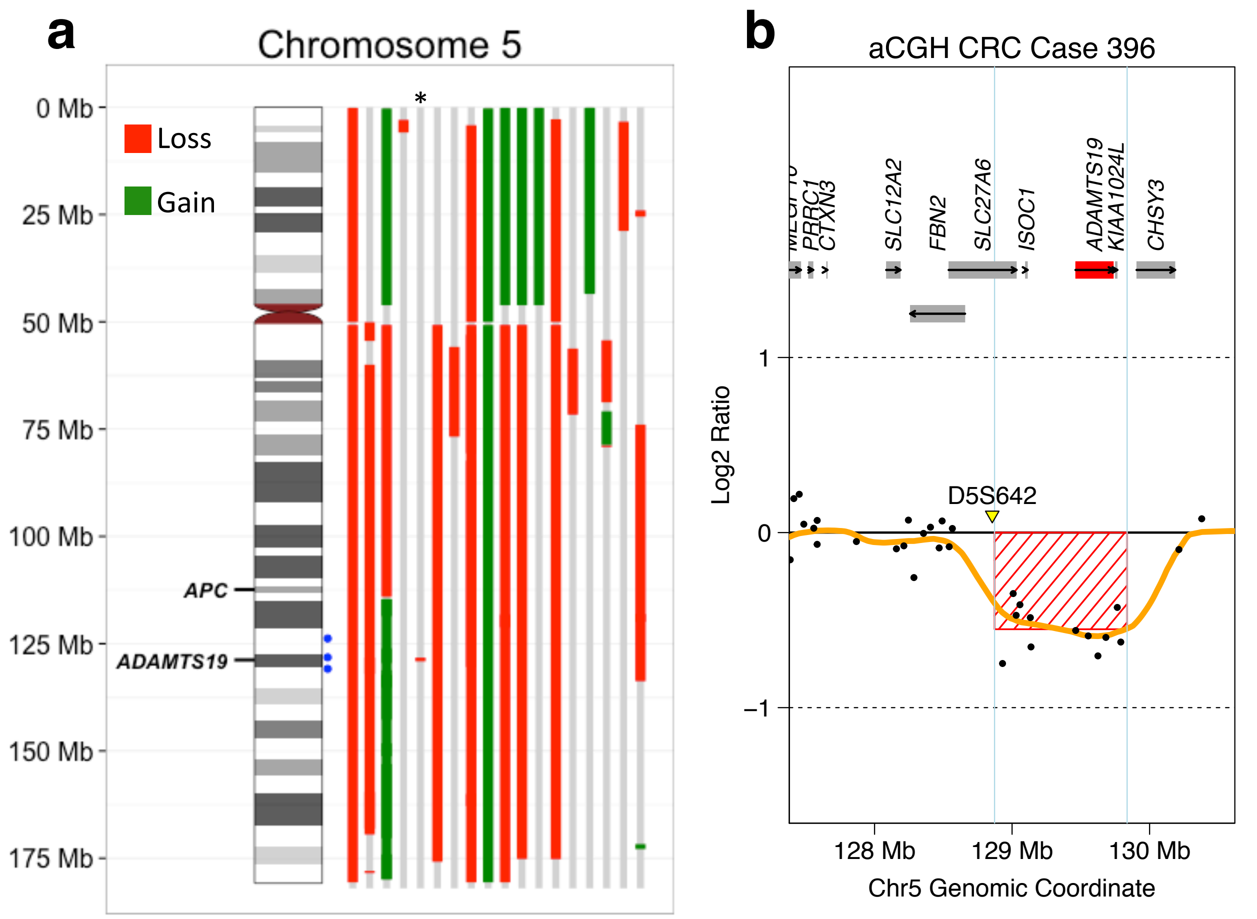

Figure S2. a) Copy number alterations in chromosome 5 analyzed by microallelotyping (in 53 cases) and array CGH (in 50 cases). Only the 18 cases with alterations in the array CGH, out of the 50 cases analyzed, are shown (vertical bars on the right side of the ideogram). Microallelotyping using markers D5S2098, D5S642 and D5S2057 (blue dots, from centromere to telomere), revealed no association between LOH (6/53, 11.3%) and hypermethylation (15/53, 28.3%) (*p*=1.0, Fisher's exact test). aCGH revealed loss of *ADAMTS19* locus in 9 cases (17%), 8 of which also showed loss of APC (OR=198.2, $p=1.5 \times 10^{-7}$). The asterisk indicates case 396 that exhibited a focal loss of 860 Kb encompassing the genes *SLC27A6*, *ISOC1* and *ADAMTS19*. In red, regions with losses, in green regions with gains, and in grey regions with no change. b) Detailed view of the region of loss in case 396. Genes in this region are depicted in the upper part of the graph, with arrows indicating the sense of transcription. aCGH probes are indicated by black dots. The orange line indicates the triangular moving average in 1Mb windows. The dashed red rectangle represent the region lost detected by the ADM-2 algorithm. The triangle points the location of the D5S642 marker, that indicated perfect heterozygosis in case 396.

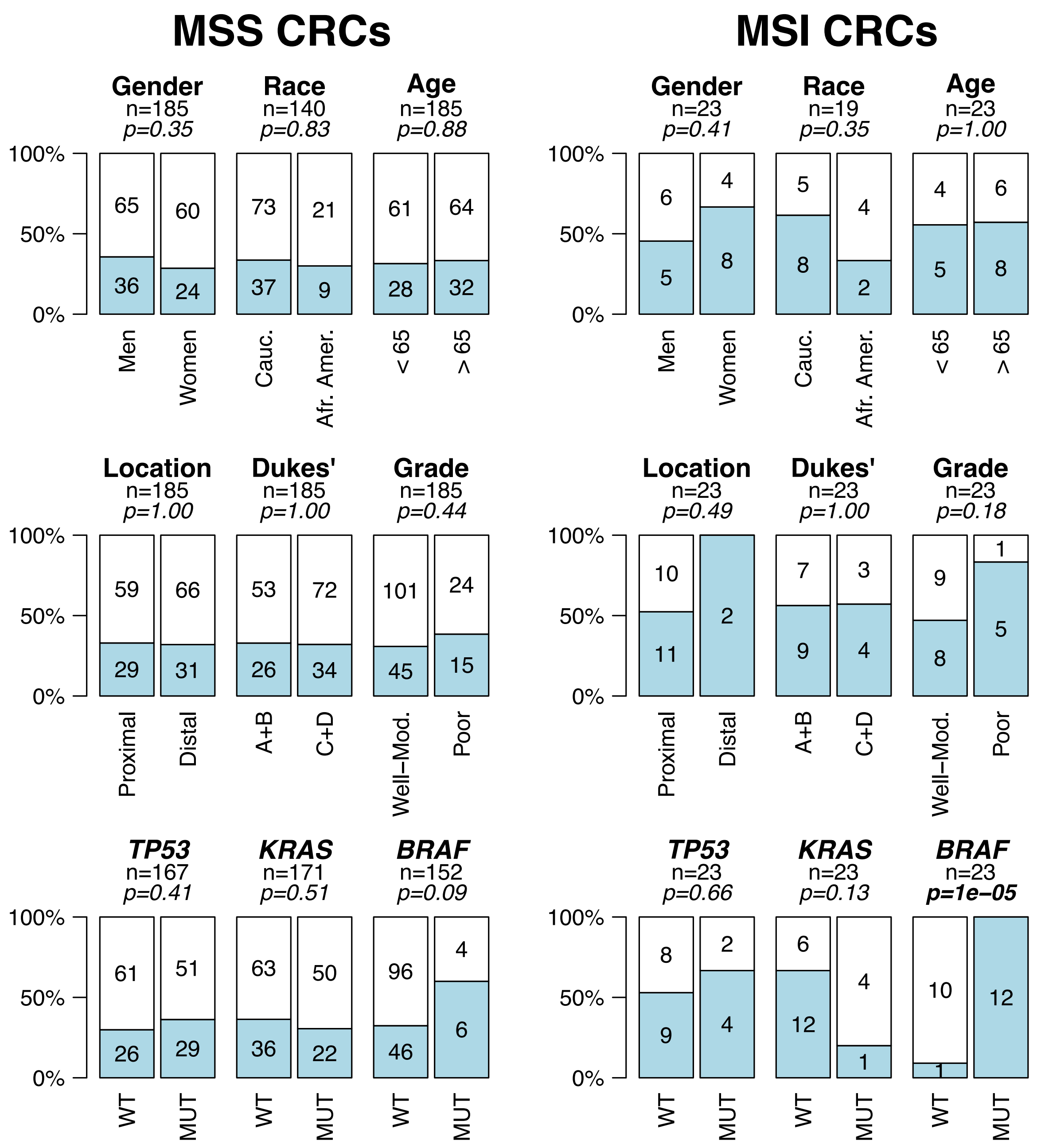

Figure S3. *ADAMTS19* hypermethylation and CRC tumor genotype and phenotype according to MSI status. In blue, methylated cases. In white, unmethylated cases. P-values are calculated by Fisher's exact test.

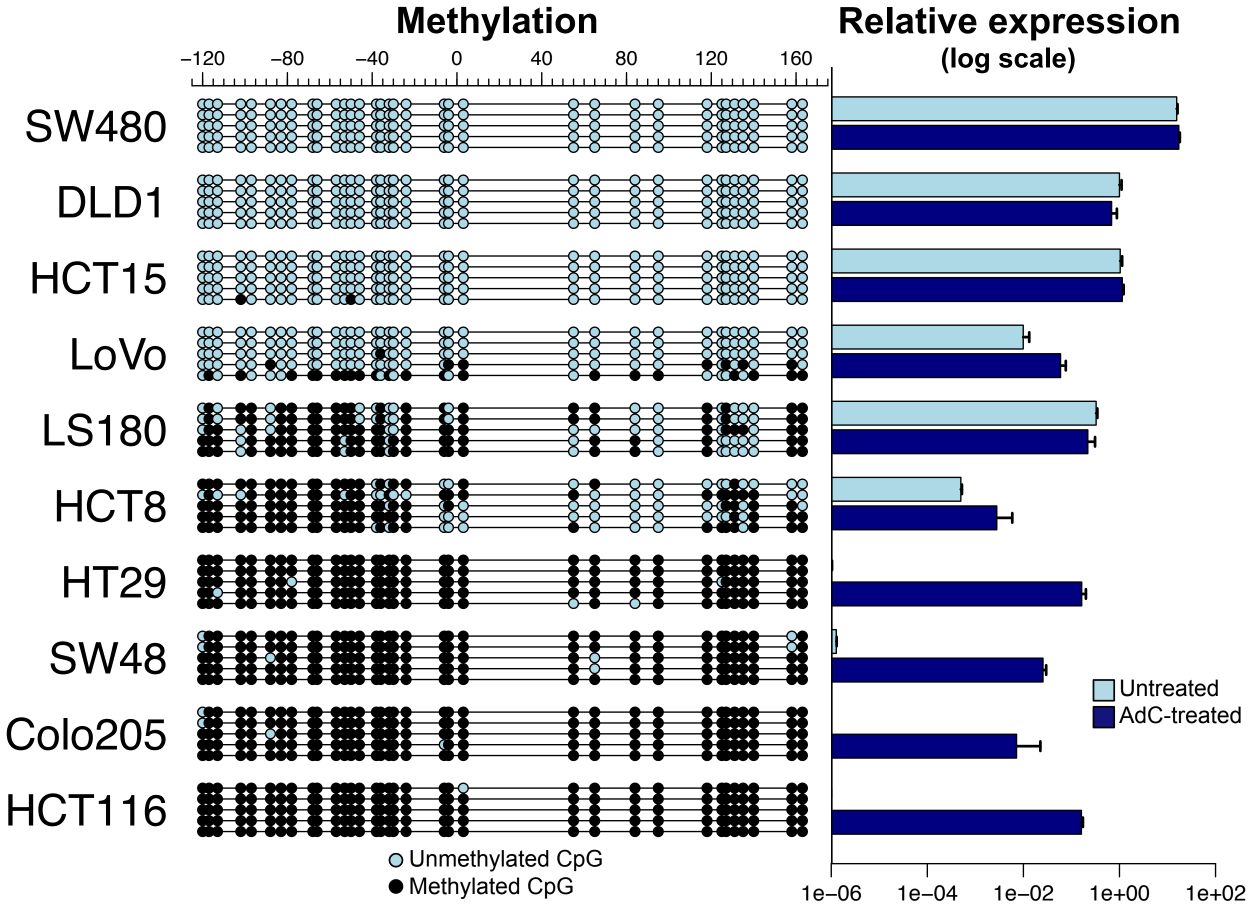

Figure S4. Methylation of *ADAMTS19* CGI associates to transcriptional levels in cell lines. On the left panel, bisulfite sequencing *ADAMTS19* CGI in 10 CRC cell lines (ordered by their average methylation level) revealed a strong inverse correlation with the gene transcriptional levels measured by qPCR (light blue bars, right panel). Expression levels were normalized using *GAPDH* and *TPT1* house keeping genes, and referenced to the level of DLD1. After 48h of treatment with 1μM 5-aza-2-deoxycytidine (AdC), *ADAMTS19* expression is restored in the four most methylated cell lines, HT29, SW48, Colo205 and HCT116 (dark blue bars, right panel).

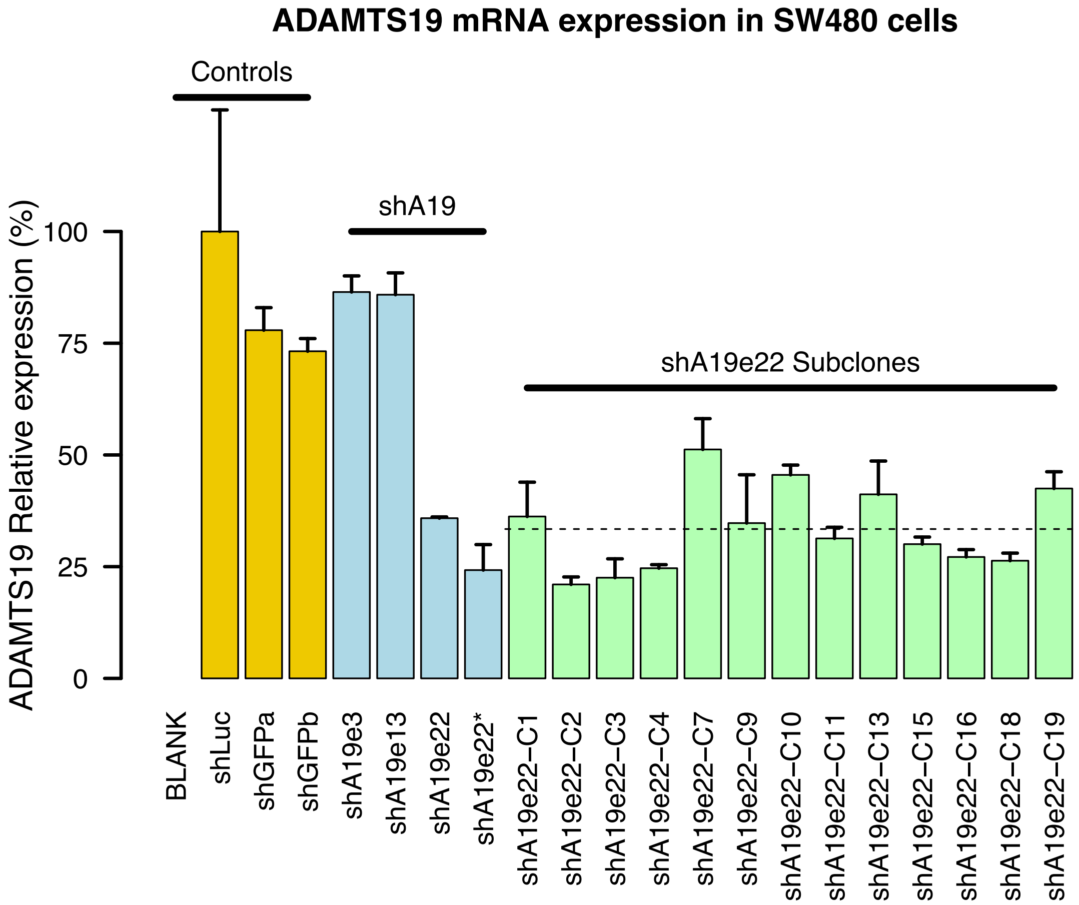

Figure S5. Transcriptional silencing of *ADAMTS19* in SW480 cells using different shRNA constructs and combinations. In orange, the control cell line SW480 transfected with a construct targeting the luciferase gene or two constructions targeting GFP (shGFPa and shGFPb) (both GFP and luciferase are not present in SW480). In blue, SW480 cells transfected with three different shRNA constructs against exons 3 (shA19e3), 13 (shA19e13) and 22 (sh19e22) of *ADAMTS19*. An additional control of SW480 transfected with this construct in the absence of puromicin was included to analyze the effect of the antibiotic (shA19e22*). In green, 13 individual subclones of SW480 transfected with shA19e22. The dashed horizontal line indicates the average expression level in these subclones. Error bars indicate the standard deviations.

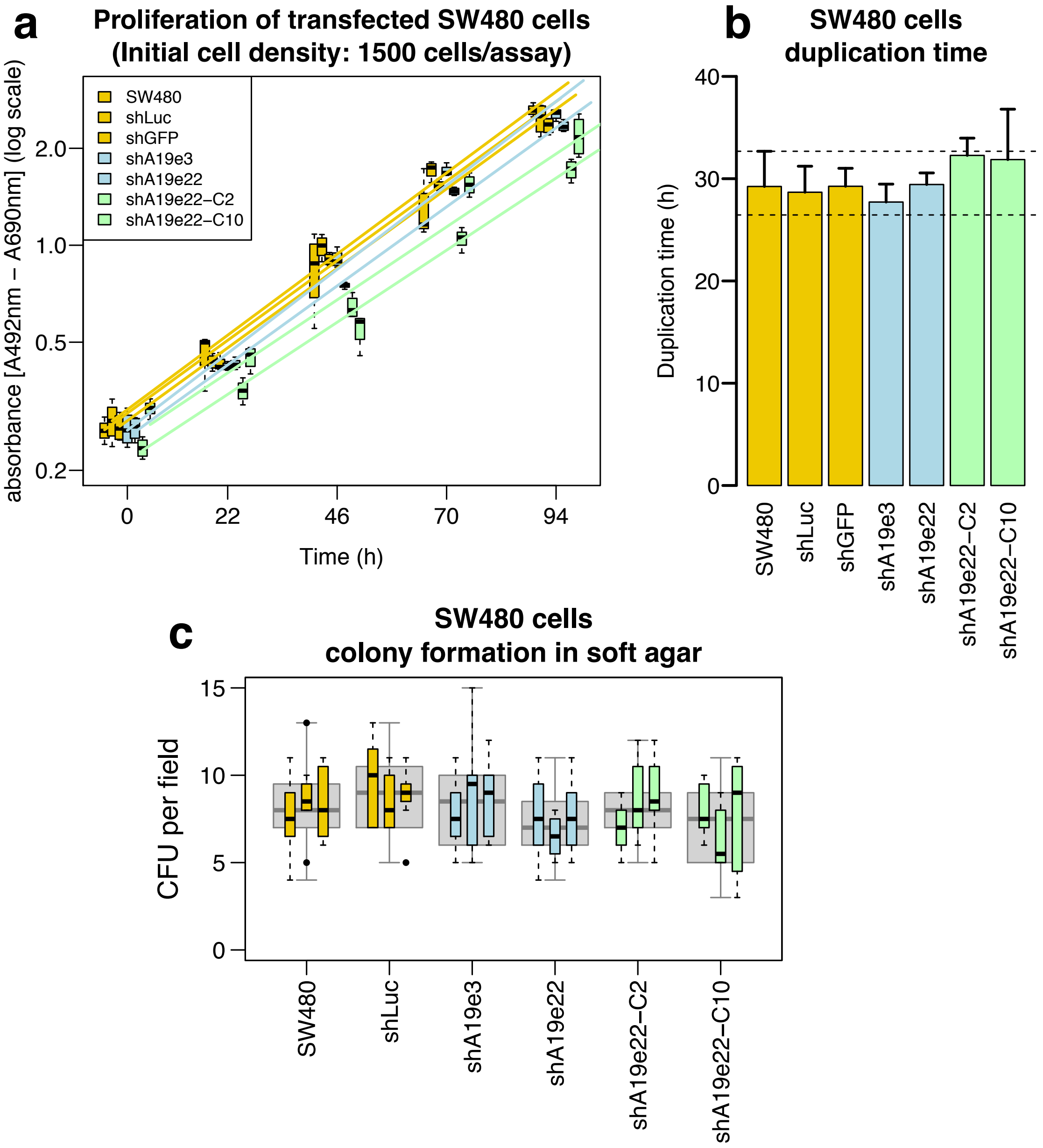

Figure S6. Silencing of *ADAMTS19* in SW480 cells does not affect their proliferation rate (panels a and b), nor their capability of forming colonies in soft agar (panel c). In orange, the control cell lines SW480 without transfection, or transfected with shRNA constructs targeting GFP (shGFP) or luciferase gene (shLuc). In blue, SW480 cells transfected with shRNA constructs targeting the exon 13 (shA19e13) or exon 22 (shA19e22) of *ADAMTS19*, and in green two individual subclones of these last cells with high level (subclone C2) or low level (subclone C10) of silencing (see figure S5). No statistical significant differences were found.

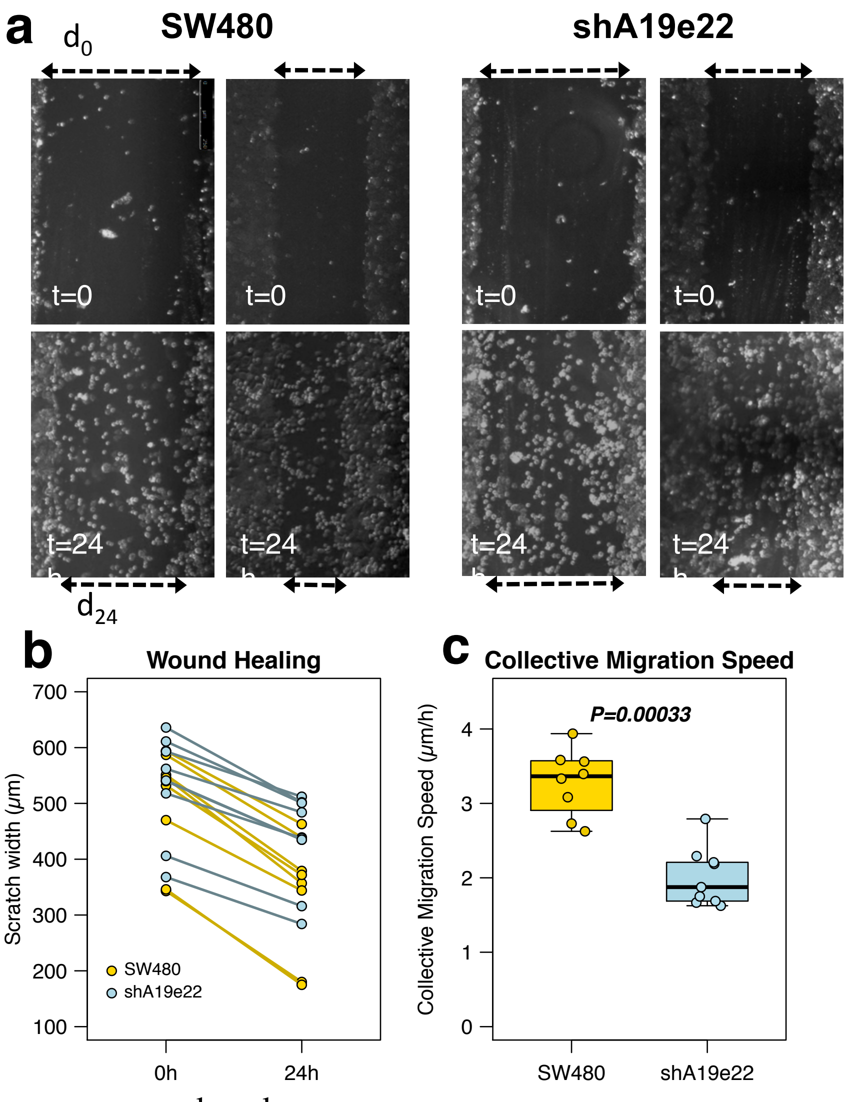

$$\mathrm{CMS} = \frac{d_0 - d_{24}}{24 \times 2}\,\mu\mathrm{m/h}$$

Figure S7. Representative images of the wound healing assays in SW480 (panel a, left) and SW480+shA19ex22 cells (panel a, right). SW480 cells generated a population of floating cells that colonized the scratches regardless of the shRNA transfection. Average scratch width was measured at time 0 (d_0, upper dashed arrows, panel a) and after 24h of culture (d_{24}, lower dashed arrows, panel b) not taking into account the individual cells within the scratch. Non-transfected SW480 cells covered the scratch faster than the shA19e22-transfected cells (panel b). Collective migration speed (CMS) was calculated using the formula shown in the figure. Cells transfected with shA19e22 reduced in 40% their collective migration speed (p=0.00033, Wilcoxson exact test, panel c). Multivariate regression confirmed that shA19e22-transfected cells reduced its CMS in 1.3μm/h ($p=2.8\times10^{-5}$), while the scratch width at time 0 did not affect CMS (p=0.64).

Table S1. Oligonucleotides used in this study.

Oligo	Sequence
NotI-G	5'-GACTGCGTAGGGGCCGCG-3'
MseI-C	5'-GATGAGTCCTGAGTAAC-3'
MseI-CA	5'-GATGAGTCCTGAGTAACA-3'
MseI-CG	5'-GATGAGTCCTGAGTAACG-3'
P28	5'-GCGTGCGCCGGGCGAGAAGC-3'
P29	5'-CGCTGGGATGGCTGGAAAGG-3'
P69	5'-ATGCGCCTGACTCACATCT-3'
P44	5'-TTGGTTGTGGGTTTGTTTTG-3'
P46	5'-CCCCTTAACCTACACCTCAAAC-3'
P16	5'-GGTAGATAAAGGGTTTGGGTAAAT-3'
P45	5'-CCCACTAAACCCTACTCTCCAC-3'
PB176	5'-AGAGCAGACAAGTGGCCTGT-3'
PB177	5'-CCTGGGTCTGGTAGAGAGGA-3'
Exon3-F	5'-GATCCCCGCTCAATGAGGACTTCATATTTTCAAGAGAAATATGAAGTCCTCATTGAGCTTTT-3'
Exon3-R	5'-TCGAAAAAGCTCAATGAGGACTTCATATTTCTCTTGAAAATATGAAGTCCTCATTGAGCGGG-3'
Exon13-F	5'-GATCCCCGCTTATAGTGTTAGAACTTCCTTCAAGAGAGGAAGTTCTAACACTATAAGCTTTT-3'
Exon13-R	5'-TCGAAAAAGCTTATAGTGTTAGAACTTCCTCTCTTGAAGGAAGTTCTAACACTATAAGCGGG-3'
Exon22-F	5'-GATCCCCGCTGAAGACTGTGAGGATTATTTCAAGAGAATAATCCTCACAGTCTTCAGCTTTT-3'
Exon22-R	5'-TCGAAAAAGCTGAAGACTGTGAGGATTATTCTCTTGAAATAATCCTCACAGTCTTCAGCGGG-3'
